# A Complex Relationship: Psychosocial Stress, Pollution, and Health

**DOI:** 10.1289/ehp.117-a407a

**Published:** 2009-09

**Authors:** Tina Adler

**Affiliations:** **Tina Adler** first wrote for *EHP* about the Clinton–Gore environmental agenda in 1993. She is a member of the National Association of Science Writers and the American Society of Journalists and Authors

In recent years, a growing body of work has shown that psychosocial stress may exacerbate susceptibility to the adverse effects of pollutants such as lead, polychlorinated biphenyls, and combustion emissions. To accurately measure and evaluate the effects of stress on people’s susceptibility to pollutants, researchers need to rely on the tools and findings of both social epidemiology and environmental health science, according to a review of the research to date **[*EHP*** **117:1351–1358; Clougherty and Kubzansky]**. The authors offer specific recommendations for how researchers can combine techniques from these fields to investigate the links between stress, pollution, and health.

In the authors’ own earlier studies, they found that stress seemed to exacerbate effects of pollution, which suggests that stress increases susceptibility to environmental exposures. However, they also noted that the interaction between stress and pollution was no longer evident beyond a certain range of exposure, a phenomenon they refer to as the saturation effect. For example, if air pollution levels are very high, stress may have no additional effect on the likelihood of asthma symptoms occurring, and vice versa.

It’s also important to pay attention to differences in spatial patterns of social and physical exposures. As an example, the authors write, “spatial epidemiologists are challenged to differentiate health effects of traffic-related pollution from those of spatially correlated noise, stress, or poverty.” It can be particularly difficult to separate the effects of different exposures if they affect the same health outcomes. Moreover, not every individual within this sample neighborhood would necessarily experience high levels of stress, nor would every individual receive the same traffic-related pollution exposures, which the authors point out vary dramatically within 50–200 m of major roadways.

To understand the combined effects of stress and pollutant exposures, timing is everything because acute and chronic stress can produce different results. Acute stress can produce “fight-or-flight” responses that might counterbalance the effects of pollution—for example, stress-induced bronchodilation might temporarily reduce or mask bronchial constriction caused by air pollution. Chronic stress is more likely to gradually weaken the immune system, increasing susceptibility to pollution-related illness. Stress is also multidimensional; it includes the stimulus that poses the challenge, the person’s appraisal of the stressor, and finally the psychological and physiological response.

When measuring stress, researchers must consider what stage of the stress experience they are observing. They also must track the relative timing of study participants’ exposures to determine whether the stress occurred before, after, or during the pollutant exposure. In all, the authors write, “These topics are exceedingly complicated, and accurately characterizing both social and physical exposures is a significant challenge, one which must be performed carefully . . . before analyzing and interpreting interactions.”

